# Ultrafast internal dynamics of chiral domain walls probed by time-resolved x-ray resonant magnetic scattering

**DOI:** 10.1063/4.0001223

**Published:** 2026-10-15

**Authors:** Dmitriy Ksenzov, Flavio Capotondi, Nicolas Jaouen, Raphael Gruber, Vincent Cros, Mathias Kläui, Matteo Pancaldi, Emanuele Pedersoli, Nicolas Reyren, Christian Gutt

**Affiliations:** 1 Universität Siegen, Walter-Flex-Straße 3, 57072 Siegen, Germany; 2 Elettra Sincrotrone Trieste, Strada Statale 14, km 163.5, 34149 Basovizza, Trieste, Italy; 3 Department of Molecular Sciences and Nanosystems, Ca' Foscari University of Venice, Venice, Italy; 4 Synchrotron SOLEIL, Saint-Aubin, Boîte Postale 48, 91192 Gif-sur-Yvette Cedex, France; 5 Institut für Physik, Johannes Gutenberg-Universität Mainz, 55099 Mainz, Germany; 6 Laboratoire Albert Fert, CNRS, Thales, Université Paris-Saclay, 91767 Palaiseau, France

## Abstract

We measure the picosecond dynamics of labyrinthine stripe domains with chiral Néel-type domain walls using time-resolved x-ray resonant magnetic scattering (XRMS). At the stripe-domain wavevector, the helicity-summed signal shows ultrafast demagnetization and recovery together with a weak oscillatory contribution attributed to laser-launched coherent surface phonons. In contrast, the dichroic signal, which is sensitive to the in-plane magnetization within the Néel domain walls, exhibits a pronounced oscillatory response. Interestingly, the observed frequency of domain wall dynamics decreases with increasing pump fluence. We associate this frequency decrease with a transient softening of the domain-wall restoring potential following optical excitation. This finding highlights that time-resolved XRMS isolates internal domain-wall dynamics and provides reciprocal-space access to wall-specific degrees of freedom in disordered stripe textures on picosecond time scales.

Magnetic multilayers with perpendicular magnetic anisotropy (PMA) can host chiral Néel domain walls stabilized by the interfacial Dzyaloshinskii–Moriya interaction (i-DMI) and support current-driven domain-wall motion.^1–3^ The same interactions can also stabilize topological spin textures such as skyrmions in multilayer systems.^4,5^

Ultrafast optical excitation can modify magnetic order on sub-picosecond time scales, but the picosecond dynamics of chiral domain walls remain poorly explored experimentally. This regime is particularly relevant when transient heating drives the system toward the Curie temperature, softens the effective anisotropy, and modifies the domain-wall structure and stiffness.^6,7^

Furthermore, femtosecond optical pump pulses generate transient strain, change the magnetic free energy, and perturb the internal wall structure. Time-resolved magneto-optical probes are generally sensitive to a combination of these responses and do not provide the reciprocal-space selectivity needed to isolate the response of a domain pattern. In contrast, time-resolved x-ray resonant magnetic scattering (XRMS) probes Fourier components of magnetic textures and distinguishes the helicity-summed and dichroic responses by combining measurements with opposite photon helicity.^8–10^ This combination of reciprocal-space and helicity selectivity provides a means of distinguishing internal chiral domain-wall dynamics from the concurrent demagnetization and structural response. Nevertheless, ultrafast measurements exploiting this selectivity to isolate chiral domain-wall dynamics remain limited.^11,12^

In this study, we use time-resolved XRMS to measure the picosecond response of chiral domain walls following femtosecond optical excitation. By measuring the helicity-summed and dichroic signals at the stripe-domain wavevector, we separate a weak oscillatory contribution attributed to coherent surface phonons from a pronounced domain-wall-sensitive oscillation. The frequency of the latter decreases systematically with increasing pump fluence, showing that the characteristic frequency of the internal domain-wall response depends on the excitation conditions. We discuss this behavior below as being consistent with a fluence-dependent softening of the effective restoring potential governing the internal wall dynamics.^1,12,13^

Time-resolved soft XRMS experiments were performed at the DiProI endstation of the FERMI free-electron laser (FEL) facility in Trieste, Italy.^14–16^ A schematic of the experimental geometry is shown in Fig. 1(a). The magnetic multilayers were measured in reflection geometry at an incidence angle of θ=45°. Ultrafast excitation was provided by a nearly collinear infrared pump pulse (λ_L_ = 790 nm) with linear horizontal (LH) polarization and a pulse duration of ∼60 fs. The absorbed pump fluence *F* varied between 1.5 and 4.5 mJ cm^−2^. The pump spot on the sample surface was ∼600 × 670 *μ*m^2^.

**FIG. 1.**
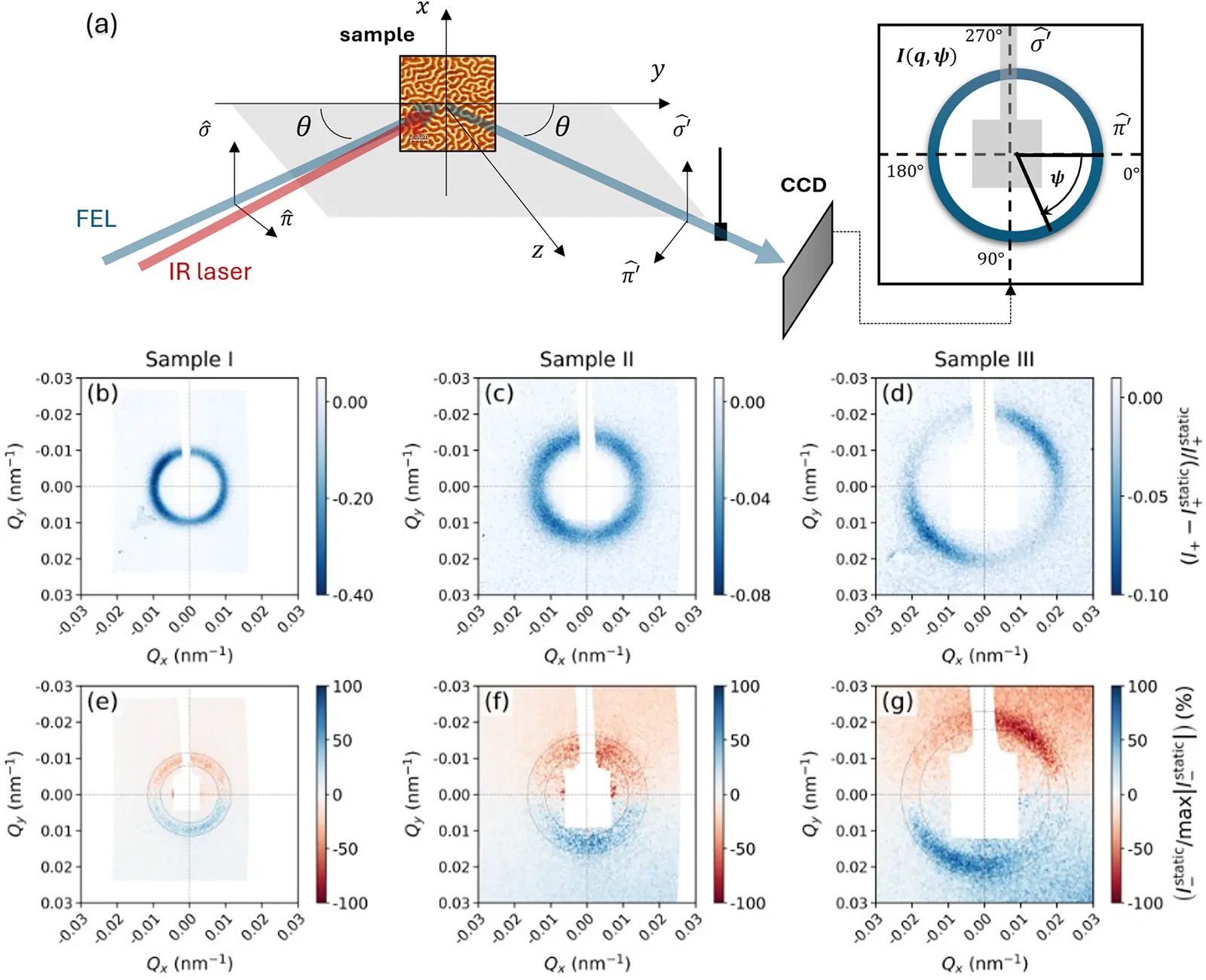
(a) Schematic of the time-resolved XRMS experiment. Infrared (IR) pump pulses ( $\lambda_{L}=790\text{ nm}$) excite the multilayer. Circularly polarized FEL pulses tuned to the Fe M_2,3_ edge ( λ=22.75 nm) probe the response in reflection geometry at θ≈45°. (b)–(d) Normalized differential sum signal, $\left(I_{+}^{\text{pumped}}-I_{+}^{\text{static}}\right)/I_{+}^{\text{static}}$, at a pump–probe delay of 0.5 ps for samples I, II, and III, respectively. The scattering ring corresponds to the mean stripe-domain periodicity; its intensity decreases after pumping due to reduced magnetic scattering contrast. (e)–(g) Normalized static dichroic signal, $I_{-}^{\text{static}}$, sensitive to in-plane domain-wall magnetization. According to the scattering geometry [panel (a)], samples I and II show Néel-type walls, while sample III exhibits mixed Néel and Bloch character.

The FEL probe pulses had a duration of approximately 60 fs and were delivered at a repetition rate of 50 Hz. The probe wavelength was 22.75 nm, corresponding to the Fe M_2,3_ absorption edge. The probe beam was focused to a spot size of approximately 320 × 290 *μ*m^2^. The pump–probe delay *t* was varied up to 600 ps using an optical delay stage. Scattered extreme-ultraviolet radiation was recorded in reflection geometry using a two-dimensional charge-coupled-device (CCD) detector placed at 150, 100, and 75 mm from samples I, II, and III, respectively. The sample-to-detector distances were selected according to the different magnetic domain periodicities, optimizing the corresponding magnetic scattering rings' momentum resolution within the accessible detector area. The specular beam was blocked by a beamstop. At each delay, patterns were averaged over 500 pump–probe shots.

Measurements were performed on three different Si/SiO_2_/[HM/CoFeB/MgO]_n_/Ta multilayers: sample I, [Ir(6.6 nm)/CoFeB(0.7 nm)/MgO(2.0 nm)]_15_; sample II, [W(5.0 nm)/CoFeB(0.8 nm)/MgO(2.0 nm)]_7_; and sample III, [Ir(6.6 nm)/CoFeB(0.7 nm)/MgO(2.0 nm)]_7_. All multilayers exhibited PMA and formed labyrinthine stripe domains in the remanent state at room temperature (∼300 K). The stripe-domain periodicity was determined from the radius of the magnetic scattering ring. Due to the different multilayer structure, the magnetic domain peak appears in the XRMS signal at different wave vectors [Figs. 2(b)–2(d)]: Q_M_ ≈ 0.010 nm^−1^ (sample I), Q_M_ ≈ 0.0145 nm^−1^ (sample II), and Q_M_ ≈ 0.021 nm^−1^ (sample III), corresponding to stripe-domain periods of approximately 630, 430, and 300 nm, respectively.

**FIG. 2.**
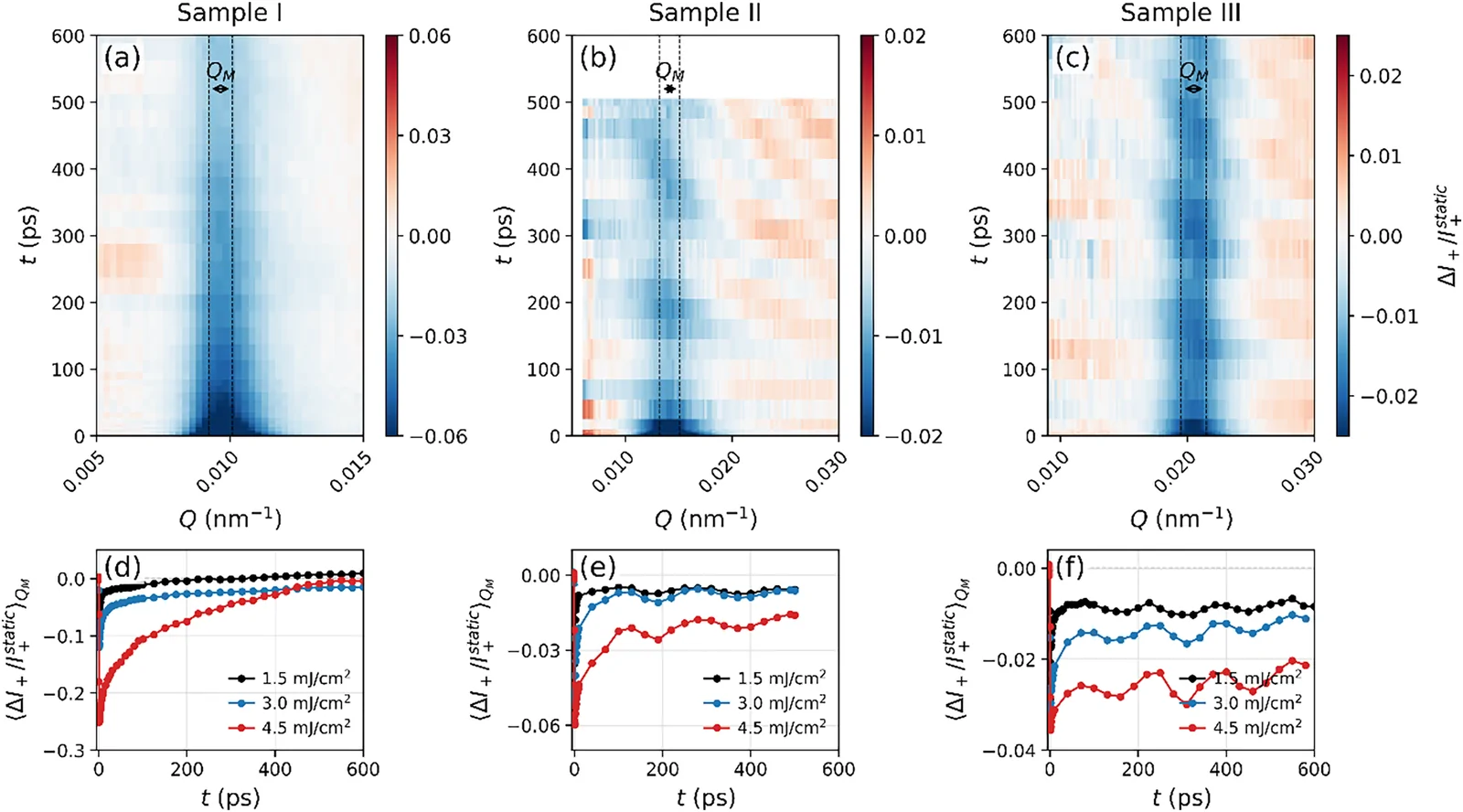
(a)–(c) Time–momentum maps of $\Delta I_{+}/I_{+}^{\text{static}}$ at *F* = 3.0 mJ cm^−2^ for samples I (a), II (b), and III (c), respectively. (d)–(f) Corresponding momentum-integrated responses averaged around $Q_{M}$, shown as a function of pump–probe delay for *F* = 1.5 (black), 3.0 (blue), and 4.5 mJ cm^−2^ (red).

In resonant magnetic scattering, the total scattering amplitude is written as the sum of structural and magnetic terms,^8^

$$f=f_{0}+f_{m},$$ (1)

where $f_{0}$ and $f_{m}$ are the charge and magnetic scattering amplitudes. Using left- and right-circularly polarized probe pulses, the measured intensities are $I_{CL}$ and $I_{CR}$. We define the helicity-summed and dichroic signals as

$$\;I_{+}=I_{CL}+I_{CR},\;I_{-}=I_{CL}-I_{CR}.$$ (2)

To leading order,^8,9^

$$\;I_{+}\propto {\left|f_{0}\right|}^{2}+{\left|f_{m}\right|}^{2},\;I_{-}\propto \;Im\left(f_{0}f_{m}^{*}\right),$$ (3)

where the dichroic term arises from charge–magnetic interference. Here, Im() denotes the imaginary part. In the present reflection geometry, the dichroic signal $I_{-}$ is sensitive to the in-plane magnetization within both Néel- and Bloch-type domain walls. The two wall configurations produce different azimuthal symmetries of the dichroic scattering, rotated by 90° with respect to each other in accordance with the scattering geometry shown in Fig. 1(a).^10^

Figures 1(b)–1(d) show the normalized differential sum signal $\Delta I_{+}/I_{+}^{\text{static}}$, defined as $\left(I_{+}^{\text{pumped}}\left(t\right)-I_{+}^{\text{static}}\right)/I_{+}^{\text{static}}$, at *t* = 0.5 ps for samples I–III. The reduced ring intensity at *Q_M_* is consistent with the loss of magnetic scattering contrast caused by optically induced ultrafast demagnetization.^7,11,17^ The dichroic signal is sensitive to the chiral magnetic contribution. The static dichroic patterns in Figs. 1(e)–1(g), $I_{-}^{\text{static}}/\text{max}\left|I_{-}^{\text{static}}\right|$, show an angular modulation determined by the in-plane domain-wall magnetization and its Fourier components at *Q_M_*, with the symmetry observed for samples I and II being consistent with Néel-type domain walls.^9,18^

For samples I and II, the maxima of static dichroic contrast in the unpumped remanent state occur at 90° and 270°, consistent with Néel-type walls with a unique chirality throughout the multilayers. In sample III, the dichroic extrema are shifted to 135° and 315°, indicating a deviation from the simple Néel-type azimuthal symmetry. This shift may reflect either a more complex wall texture^19,20^ or the influence of the stripe-pattern orientation on the measured dichroic contrast.

Figures 2(d)–2(f) summarize the dynamics of $\Delta I_{+}/I_{+}^{\text{static}}$ around the magnetic ring associated with *Q_M_*. The traces were obtained by averaging over *Q_M_* in the range 0.0092–0.0101 nm^−1^ for sample I, the time–momentum map in Fig. 2(a); in the range 0.0132–0.0151 nm^−1^ for sample II, the time–momentum map in Fig. 2(b); and in the range 0.0195–0.0215 nm^−1^ for sample III, the time–momentum map in Fig. 2(c), where the time–momentum map was constructed considering the temporal dependence of the azimuthal average as a function of Q of the XRMS diffraction pattern.

To explain the dynamics reported in Fig. 2, we expand the structural and magnetic amplitudes to first order as $f_{0}\left(t\right)=f_{0}+\delta f_{0}(t)$ and $f_{m}\left(t\right)=f_{m}+\delta f_{m}\left(t\right)$.^8,9^ For the helicity-summed intensity $I_{+}$, the charge–magnetic interference term is odd under helicity reversal and cancels. The relative change of $I_{+}$ is

$$\frac{I_{+}^{\text{pumped}}(t)-I_{+}^{\text{static}}}{I_{+}^{\text{static}}}\approx \frac{2Re\left(f_{0}^{*}\delta f_{0}\left(t\right)\right)}{{\left|f_{0}\right|}^{2}+{\left|f_{m}\right|}^{2}}+\frac{2Re\left(f_{m}^{*}\delta f_{m}\left(t\right)\right)}{{\left|f_{0}\right|}^{2}+{\left|f_{m}\right|}^{2}}.$$ (4)

Here, Re() denotes the real part. The first term on the right-hand side of Eq. (4) represents a structural contribution associated with lattice displacements and strain. This contribution is weak and shows a small oscillatory component with an amplitude below approximately 0.5% over the studied fluence range. The oscillation period is in the tens to hundreds of picoseconds and does not depend strongly on pump fluence [see Figs. 2(b) and 2(c)]. The observed modulation is consistent with optically launched coherent surface phonons reported previously for metallic films and multilayers.^21^ The second term describes the magnetic contribution at *Q_M_*, which is dominated by ultrafast demagnetization followed by recovery of magnetization.^7,17^

The relative visibility of the weak oscillatory contribution differs among the three samples. For sample I, the helicity-summed signal exhibits a prompt decrease of approximately 10%–25%, depending on pump fluence. This strong magnetic response masks much the weaker structural modulation, and no clear oscillation, attributable to lattice dynamics, can be resolved in Figs. 2(a) and 2(d). In contrast, an oscillatory contribution is clearly visible in the helicity-summed responses of samples II and III [Figs. 2(b), 2(c), 2(e), and 2(f)]. For these two samples, the initial signal reduction amounts to only approximately 2%–6%, allowing the weak oscillatory contribution to remain visible on top of the magnetic background.

Figures 3(a) and 3(c) show time–azimuth maps of the normalized dichroic signal, $I_{-}(t,\psi )/I_{-}^{\text{static}}$, for samples I and II at a pump fluence of *F* = 3.0 mJ cm^−2^, respectively. The maps were obtained by averaging over Q in the ranges 0.0092–0.0101 nm^−1^ around *Q_M_* for sample I and 0.0132–0.0151 nm^−1^ for sample II. The dichroic signal exhibits a pronounced maximum at ψ ≈ 270°, consistent with a Néel-type wall configuration, and this azimuth is used to track the chiral dynamics.^9,10,12^ The maps cover the azimuthal range from 180° to 360°. Figures 3(b) and 3(d) present the corresponding azimuthally integrated dichroic signals as a function of pump–probe delay for fluences *F* = 1.5, 3.0, and 4.5 mJ cm^−2^. In both samples, the transient response displays damped oscillatory behavior over the first few hundred picoseconds. The oscillation amplitude decays with delay, so that only the first few maxima are well resolved. The two samples exhibit distinctly different oscillation frequencies. In contrast, no clear oscillatory behavior is observed in the dichroic response of sample III.

**FIG. 3.**
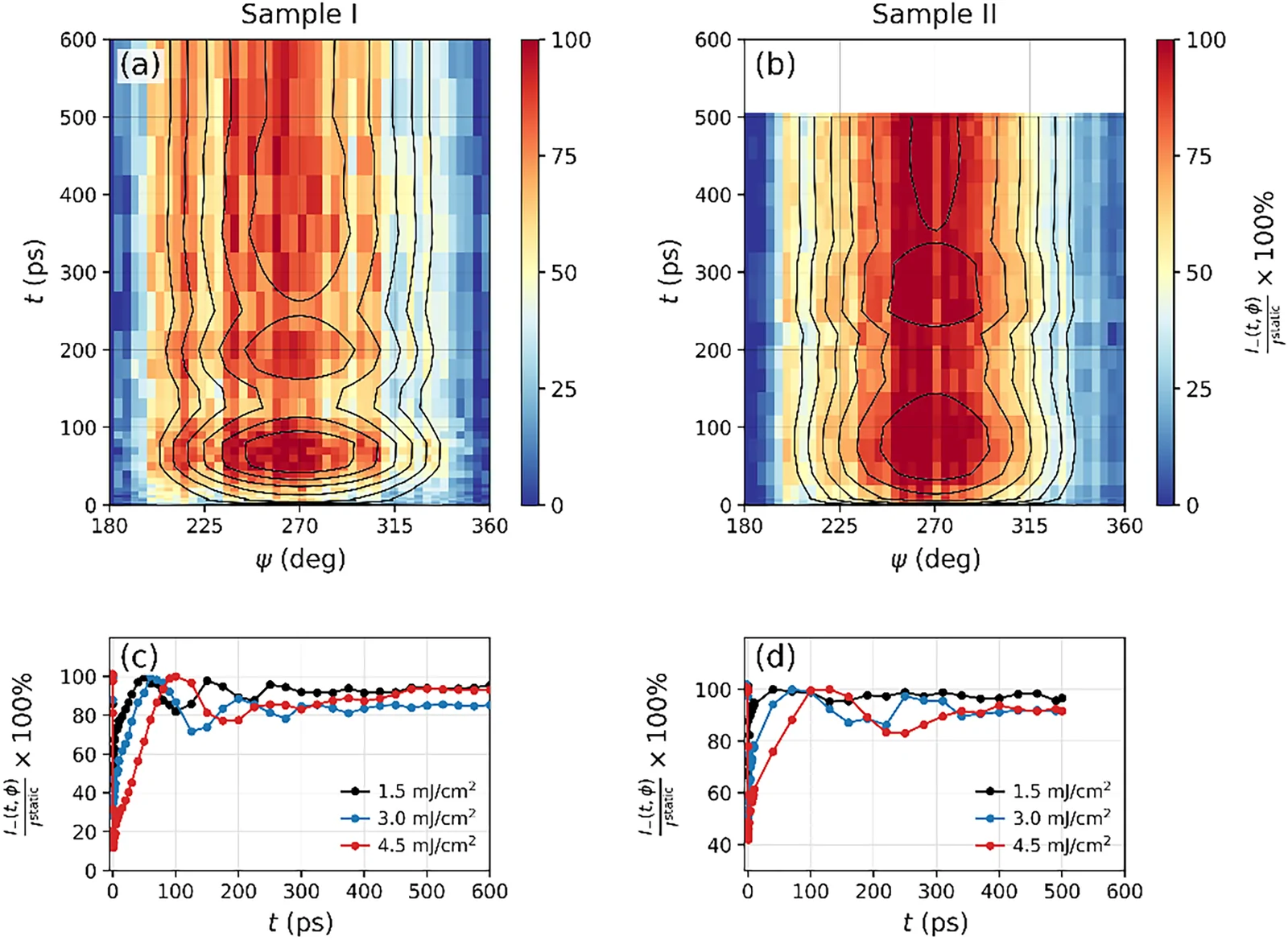
(a) and (c) Time–azimuth maps of the normalized dichroic response $I_{-}(t,\psi )/I_{-}^{\text{static}}$, for samples I (a) and II (c) at a pump fluence of *F* = 3.0 mJ cm^−2^. (b) and (d) Corresponding azimuthally integrated transient dichroic responses shown as a function of pump–probe delay for pump fluences of 1.5 (black), 3.0 (blue), and 4.5 mJ cm^−2^ (red).

Using the standard one-dimensional Walker domain-wall profile,^13^ the in-plane wall magnetization scales as $m_{∥}\left(x,t\right)\propto M_{s}\left(t\right)\text{sech}\left(x/\Delta \left(t\right)\right)\text{ cos }\varphi \left(t\right),$ where $M_{s}$ is the saturation magnetization, Δ the domain-wall width, and φ the internal wall angle. Its Fourier component at Q_M_ yields, up to a numerical prefactor, a form factor proportional to $\Delta \left(t\right)\text{sech}\left({\pi Q}_{M}\Delta \left(t\right)/2\right)$. Since dichroic XRMS is sensitive to the in-plane wall magnetization,^9^ the dichroic scattering contribution can be written as

$$I_{-}(t)\propto M_{s}(t)\Delta (t)\text{sech}\left(\frac{\pi Q_{M}\Delta (t)}{2}\right)\text{ cos }\varphi (t).$$ (5)

The signal is thus sensitive to changes in $M_{s}\left(t\right)$, $\Delta \left(t\right)$, and φ(t),^9,22^ with the cos φ(t) term providing direct sensitivity to deviations from the equilibrium Néel configuration.

Equation (5) shows that the individual contributions of $M_{s}(t)$, Δ(t), and ϕ(t) cannot be determined independently from the dichroic signal alone. However, a modulation dominated by $M_{s}(t)$ should also affect the helicity-summed magnetic response. The much greater relative visibility of the oscillation in the dichroic signal therefore suggests that changes in $M_{s}(t)$ alone are insufficient to explain the observations. The present measurements do not allow the contributions from Δ(t) and ϕ(t) to be separated. We therefore attribute the oscillatory response to changes in the internal domain-wall configuration, without assigning it uniquely to either parameter. This interpretation is supported by earlier observations of ultrafast transient distortions of chiral Néel walls toward mixed Néel–Bloch configurations,^12^ as well as by previous reports of GHz-range domain-wall eigenmodes and flexural resonances in ferromagnetic nanostrips.^23,24^

The present multilayers were designed to stabilize chiral Néel walls through interfacial DMI. Extending the experiment to Bloch-type walls would require a different sample design, since nominally Bloch-like walls in thicker films can develop flux-closure structures with Néel-like caps near the film surfaces.^25^ Nevertheless, the same time-resolved XRMS approach could, in principle, be applied to their dynamics because their different magnetization textures are expected to give rise to distinct azimuthal symmetries of the dichroic scattering. Their dynamical responses need not be identical, since the coupling between a strain field and a domain-wall mode depends on its symmetry and spatial overlap with the magnetic eigenmode. Calculations have demonstrated symmetry-dependent magnetoelastic modes in Néel walls,^26^ while recent modeling predicts a multimode magnetoelastic response for SAW propagation across a Bloch wall.^27^ A quantitative comparison of Bloch- and Néel-wall dynamics would therefore require dedicated sample architectures and corresponding micromagnetic and magnetoelastic modeling. Such a comparison is beyond the scope of the present study.

Within a simplified one-dimensional domain-wall description,^13^ the characteristic frequency may be expected to scale with an effective restoring field. As a qualitative estimate, we write

$$\omega_{0}\left(T\right)∼\gamma \mu_{0}H_{k}\left(T\right),\;\mu_{0}H_{k}\left(T\right)\propto \frac{K_{\text{eff}}(T)}{M_{s}(T)},$$ (6)

where *T* denotes the effective transient temperature reached after optical excitation, γ is the gyromagnetic ratio, $H_{k}$ is the effective anisotropy field, and $K_{\text{eff}}$ is the effective perpendicular anisotropy. Equation (6) is used only as a qualitative scaling argument rather than as a quantitative model of the observed mode. The eigenfrequency of a domain wall in a labyrinthine multilayer may additionally depend on i-DMI, exchange stiffness, dipolar interactions between the layers, wall curvature, and pinning. Increasing pump fluence transiently modifies several of these quantities through laser-induced heating, including $M_{s}$ and $K_{\text{eff}}$.^6,7^ The measured decrease in frequency is therefore consistent with a softening of the effective restoring potential governing the wall excitation. The present measurements do not, however, allow this softening to be assigned uniquely to an individual magnetic parameter, and further experiment or computational modeling would be necessary to provide a complete microscopic description of the observed dynamic.

To quantify the chiral dynamics, the integrated dichroic traces were fitted over the full measured delay range. The non-oscillatory background was described by the three-exponential demagnetization and two-stage recovery model of Ref. 28, convolved with the experimental time resolution. The oscillatory component was represented phenomenologically by a damped cosine function, with the damping time treated as an independent fit parameter. The oscillation frequency was assumed to remain constant within the measured delay window of approximately 600 ps. This procedure yielded an oscillation frequency, *f*, for each pump fluence *F*, and the resulting f(F) values are summarized in Fig. 4 for samples I and II.

**FIG. 4.**
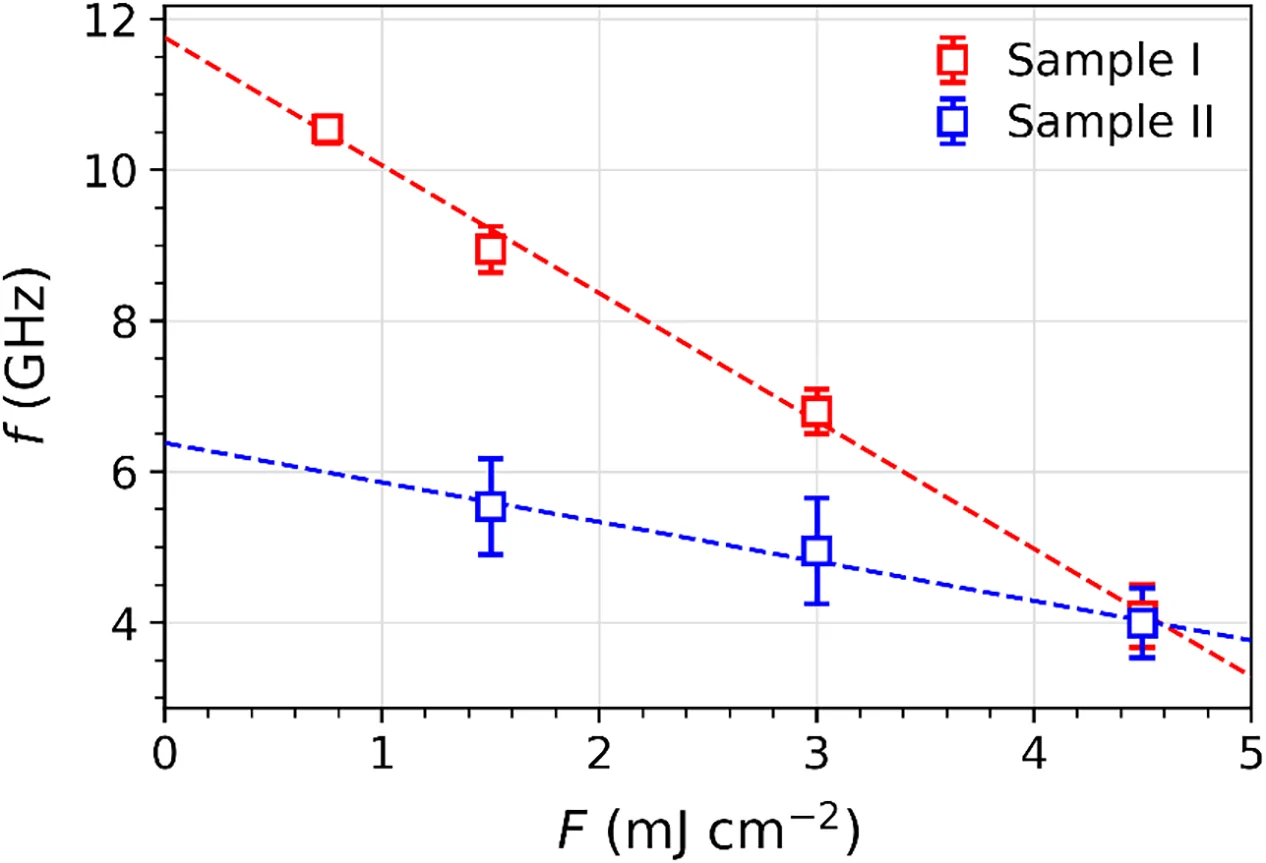
Fluence dependence of the extracted frequency for samples I (red) and II (blue). Markers show data, and dashed lines show linear fits $f\left(F\right)=f_{0}-kF$.

To parameterize the observed fluence dependence, the extracted frequencies were fitted empirically by $f\left(F\right)=f_{0}-kF$. This linear form was introduced only as a phenomenological description over the accessible fluence range, since the characteristic frequency is expected to depend on fluence-induced changes in $K_{\text{eff}}$ and $M_{s}$, as well as in the other magnetic parameters discussed above. The larger slope, *k*, obtained for sample I indicates a stronger fluence sensitivity of the wall-mode frequency. This difference likely reflects stack-dependent variations in the transient magnetic and thermal parameters that determine the restoring field, although an influence of the larger equilibrium stripe-domain period in sample I cannot be excluded.

In summary, helicity-resolved time-resolved XRMS allows us to separate the texture-averaged response of a labyrinthine domain pattern from the wall-sensitive chiral magnetic dynamics at the same domain wavevector. While the helicity-summed signal is dominated by ultrafast demagnetization and recovery, with an additional weak contribution consistent with coherent surface phonons, the dichroic signal reveals a pronounced damped oscillation that we attribute to an internal domain-wall excitation. The predominance of this oscillation in the dichroic signal suggests that a modulation of the saturation magnetization alone is insufficient to explain the response and that variations of the internal wall configuration contribute to the observed dynamics.

The decrease in the oscillation frequency with pump fluence is consistent with a transient softening of the effective restoring potential governing chiral excitation. Such softening may result from fluence-dependent changes in several magnetic parameters, including the effective anisotropy, saturation magnetization, exchange stiffness, dipolar interactions, and i-DMI; their individual contributions cannot be separated from the present measurements.

The observed dependence of the oscillation frequency on pump fluence motivates further studies of optically excited domain-wall modes and their coupling to transient strain.

See the supplementary material for two movies showing the time-resolved reciprocal-space maps measured for sample II at a pump fluence of F = 3.0 mJ cm^−2^. Supplementary Movie S1 presents the evolution of the normalized helicity-summed signal following optical excitation, including the prompt intensity reduction and subsequent oscillatory response. Supplementary Movie S2 shows the corresponding normalized dichroic response, which is sensitive to the in-plane magnetization of the chiral domain walls. The pump–probe delay is indicated in each frame. The momentum axes and color scales remain fixed throughout each movie to facilitate comparison of the spatial scattering patterns at different time delays.

## Data Availability

The data that support the findings of this study are openly available in VUO at https://doi.org/10.34965/I8821, Ref. 29 and available from the corresponding author upon reasonable request.
